# Self-Amplifying RNA Vaccines Give Equivalent Protection against Influenza to mRNA Vaccines but at Much Lower Doses

**DOI:** 10.1016/j.ymthe.2017.11.017

**Published:** 2017-12-05

**Authors:** Annette B. Vogel, Laura Lambert, Ekaterina Kinnear, David Busse, Stephanie Erbar, Kerstin C. Reuter, Lena Wicke, Mario Perkovic, Tim Beissert, Heinrich Haas, Stephen T. Reece, Ugur Sahin, John S. Tregoning

**Affiliations:** 1 BioNTech RNA Pharmaceuticals GmbH, An der Goldgrube 12, 55131 Mainz, Germany; 2 Mucosal Infection and Immunity Group, Section of Virology, Department of Medicine, St. Mary’s Campus, Imperial College London W2 1PG, UK; 3 BioNTech AG, An der Goldgrube 12, 55131 Mainz, Germany; 4 TRON GmbH, Freiligrathstraße 12, 55131 Mainz, Germany

**Keywords:** vaccine, RNA, influenza, replicon, trivalent, alphavirus, H1N1, DNA

## Abstract

New vaccine platforms are needed to address the time gap between pathogen emergence and vaccine licensure. RNA-based vaccines are an attractive candidate for this role: they are safe, are produced cell free, and can be rapidly generated in response to pathogen emergence. Two RNA vaccine platforms are available: synthetic mRNA molecules encoding only the antigen of interest and self-amplifying RNA (sa-RNA). sa-RNA is virally derived and encodes both the antigen of interest and proteins enabling RNA vaccine replication. Both platforms have been shown to induce an immune response, but it is not clear which approach is optimal. In the current studies, we compared synthetic mRNA and sa-RNA expressing influenza virus hemagglutinin. Both platforms were protective, but equivalent levels of protection were achieved using 1.25 μg sa-RNA compared to 80 μg mRNA (64-fold less material). Having determined that sa-RNA was more effective than mRNA, we tested hemagglutinin from three strains of influenza H1N1, H3N2 (X31), and B (Massachusetts) as sa-RNA vaccines, and all protected against challenge infection. When sa-RNA was combined in a trivalent formulation, it protected against sequential H1N1 and H3N2 challenges. From this we conclude that sa-RNA is a promising platform for vaccines against viral diseases.

## Graphical Abstract

**Figure:**
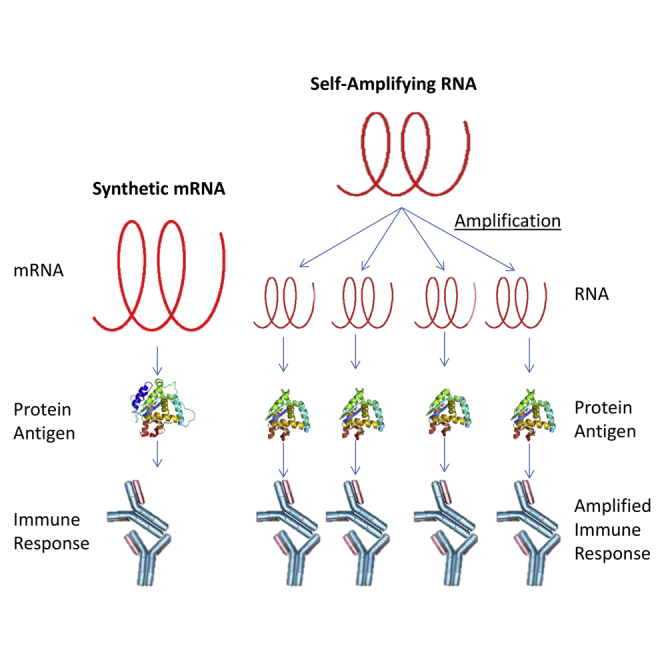

## Teaser

RNA vaccines are a promising new approach to control infectious disease. When we compared two influenza RNA vaccine platforms, we observed equivalent levels of protection using less self-amplifying RNA (1.25 μg) compared with synthetic mRNA (80 μg). In addition, a low-dose trivalent self-amplifying RNA protected against sequential H1N1 and H3N2 influenza challenges.

## Introduction

Today’s vaccine systems lack important features to be fast or flexible enough to respond to today’s challenges in pathogen control and new systems are urgently needed, especially for epidemics and pandemics. Because of increased globalization and access to rural areas, the emergence of new pathogens is increasing^1^ and new strategies are desperately needed to accelerate vaccine availability from discovery to dispensation. Nucleic acid based vaccines are promising candidates because of the speed from pathogen sequence data acquisition in the field to vaccine production. Furthermore, they may potentially require fewer regulatory tests than for instance inactivated or attenuated viruses, because nucleic acid is the invariant base product no matter the pathogen. Two nucleic acid platforms have been proposed for vaccination, namely RNA and DNA. RNA is favorable since—unlike DNA vaccines, which have to overcome two barriers to transcription,^2^ the cell and nuclear membranes—antigens can be translated from RNA vaccines as soon as they enter the cytoplasm. This increases transfection efficiency and should therefore have a knockon effect on immunogenicity. RNA vaccines further combine features of safety with fast, totally cell-free production; moreover, they induce both B and T cell responses.^3^

Using a cellular-like mRNA encoding the protein of interest (synthetic mRNA) there is an immediate translation of the antigen. Though good antiviral protection has been shown,^4^ a lot of synthetic mRNA material is needed. Scaling up from the amount of material required in small animals to humans may limit the availability of vaccine in cases of emerging epidemic and pandemic diseases. Therefore, further development of this approach is required. Modification of the synthetic mRNA molecule itself can be beneficial for immunogenicity and antigen expression, for example the development of new cap-analogs.^5^, ^6^ However, an alternative single-stranded RNA platform approach is available, that combines modification processes with increased protein translation. Self-amplifying RNA (sa-RNA) vaccines are derived from alphaviruses: positive-strand, non-segmented RNA viruses. The alphaviral genome is divided into two open reading frames (ORFs): the first ORF encodes proteins for the RNA dependent RNA polymerase (replicase), and the second ORF encodes structural proteins. In sa-RNA vaccine constructs, the ORF encoding viral structural proteins is replaced with any antigen of choice, while the viral replicase remains an integral part of the vaccine and drives intracellular amplification of the RNA after immunization.^7^

One pathogen with pandemic potential is influenza virus, which belongs to the family of *Orthomyxoviridae* and can be divided into 3 genera, influenza A, B, and C. Because of their segmented RNA genome, many subtypes exist, especially within the influenza A viruses. Mutation and recombination of different virus subtypes occurs fairly easily leading to the frequent emergence of novel strains. In humans, influenza viruses caused 3 pandemics in the 20th Century. The most recent “swine flu” pandemic in 2009 was considered a low-pathogenicity strain but still infected approximately 200 million people and caused an estimated 201,200 fatalities.^8^ The currently emerging H5N8 bird flu virus isolate further demonstrates the urgent need to flexibly adjust vaccines to highly promiscuous subtypes.^9^ The highly changeable nature of influenza virus and the history of pandemics underpin the urgent need to be ready for a new pandemic influenza virus. As the characteristics of pandemic viruses cannot be predicted, a quickly adaptable vaccine platform is needed to address this threat. Currently, most influenza vaccines are prepared from inactivated viruses, grown in embryonated chicken eggs. This can be problematic, particularly for avian-derived viruses, which may be highly pathogenic to the chicken embryo, resulting in a low titer of recoverable virus. In this respect, RNA vaccines could offer a considerable saving in time. mRNA has already been used to immunize mice, ferrets, and pigs against influenza,^4^, ^10^ and also sa-RNA has been introduced for protection against H1N1^11^ and newly emerging subtype H7N9^11^ influenza in 2013.

In the current study, we looked at the possibility of replacing the protein based seasonal influenza trivalent vaccine with an RNA vaccine. The first question is which RNA vaccine platform was best, synthetic mRNA or sa-RNA. While it is more immunogenic, the production process and stability of the sa-RNA product is more challenging because of the length of constructs. We compared synthetic mRNA and sa-RNA encoding the hemagglutinin (HA) gene from a model influenza virus strain. 64-fold less sa-RNA material was required to induce a similar level of protection, namely 80 μg mRNA versus 0.05 μg sa-RNA was needed for full survival. We then developed and tested sa-RNA encoding HA from seasonal influenza virus A and B strains and observed that they were protective both singly and as a trivalent formulation.

## Results

### sa-RNA Achieves Equivalent Protection to mRNA but Requires Less RNA

**Figure 1:**
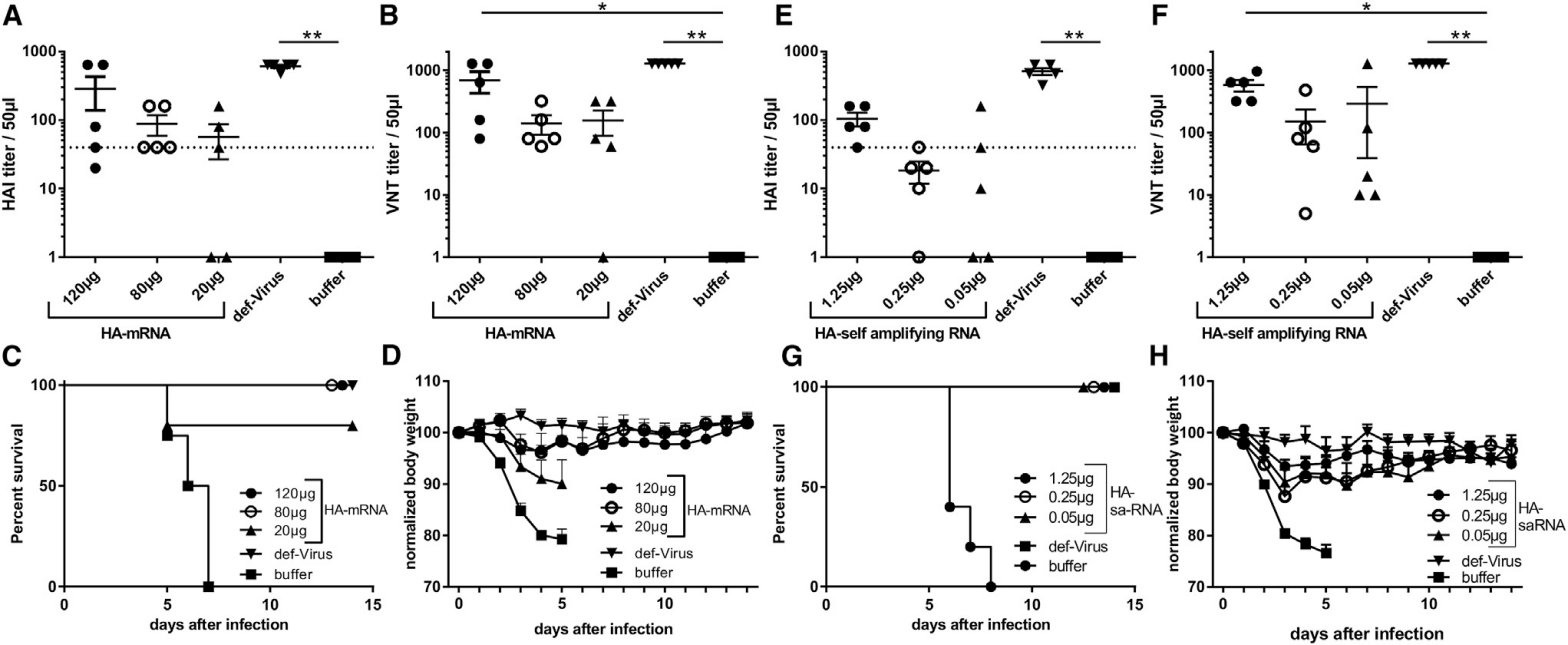
Different mRNA Vaccine Platforms Are Both Protective against Influenza A Disease in a Prime-Boost Regime, but IVT-mRNA Requires More Material BALB/c mice were immunized i.m. with 120, 80, or 20 μg H1N1/PR8-HA coding mRNA, with 5 μg of inactivated virus (def-Virus) or ringer-lactate solution only (buffer), followed by a homologous boost 3 weeks later. H1N1-specific antibody was measured by HAI (A) and VNT (B) 8 weeks after the first vaccine was administered. Animals were infected i.n. with 10-fold MLD_50_ of H1N1/PR8. Survival (C) and weight change (D) were monitored daily. BALB/c mice were immunized i.m. with 1.25, 0.25, or 0.05 μg H1N1/PR8-HA coding sa-RNA followed by a homologous boost 3 weeks later. H1N1-specific antibody was measured by HAI (E) and VNT (F) 8 weeks after the first vaccine was administered. Thereafter, animals were infected i.n. with 10-fold MLD_50_ of H1N1/PR8. Survival (G) and weight change (H) were monitored daily. Lines and points represent means and SEM of n = 5 mice. *p < 0.05 and **p < 0.001 indicate significance measured by one-way ANOVA.

To determine the protective potential of synthetic mRNA, BALB/c mice were immunized intramuscularly (i.m.) with a prime-boost regime of 120, 80, or 20 μg synthetic mRNA encoding HA from the H1N1 influenza virus A/Puerto Rico/8/1934 (H1N1/PR8), inactivated virus was used as a positive control. Antibody responses were assessed by hemagglutination inhibition (HAI) (Figure 1A) or viral neutralizing titer (VNT) (Figure 1B). Antibody responses against HA increased with increasing mRNA dose and though 80 μg induced seroconversion in all immunized animals, only 120 μg gave a VNT that was significantly greater than that in buffer-treated animals. When infected intranasally with a 10-fold lethal dose of H1N1/PR8, the 120- and 80-μg dose groups were fully protected against infection and the 20-μg dose group was partially protected (Figures 1C and 1D). In comparison, we independently performed a dose response of sa-RNA expressing the H1N1/PR8 HA antigen to analyze whether less RNA material is needed for protection compared to synthetic mRNA. Lower amounts of sa-RNA were already suspected to be potent, and therefore titration started with a lower dose. Vaccination induced an anti-H1N1/PR8 functional antibody response (Figures 1E and 1F), and a 1.25-μg dose gave a significantly greater response than did that of the buffer control. On challenge, the 1.25 μg sa-RNA group was fully protected against H1N1/PR8 infection, and the 0.25 and 0.05 μg groups were partially protected (Figures 1G and 1H). Comparing the responses (Table 1), the response in the 1.25 μg sa-RNA group was equivalent to the response in the 80 μg mRNA group, so that a 64-fold lower dose of sa-RNA than synthetic mRNA was required to give an equivalent protective response.

**Table 1:** Comparison of Responses by Different RNA Platforms

Dose	mRNA			sa-RNA		
	120 μg	80 μg	20 μg	1.25 μg	0.25 μg	0.05 μg
HAI (mean ± SD)	284 ± 325.7	88 ± 65.73	56.4 ± 66.52	104 ± 53.67	18.2 ± 14.53	42.4 ± 67.66
VNT (mean ± SD)	688 ± 581.3	140 ± 107.7	156.2 ± 152.3	576 ± 267.7	149 ± 189.6	288 ± 556.5
Weight d3 p.i.	96.7 ± 6.7	97.6 ± 2.0	93.4 ± 5.3	93.4 ± 2.9	87.6 ± 4.3	90.3 ± 5.6

HAI, hemagglutination inhibition assay titer; p.i., post-infection; VNT, viral neutralizing titer.

### sa-RNA Gives Extended Expression Compared to mRNA

**Figure 2:**
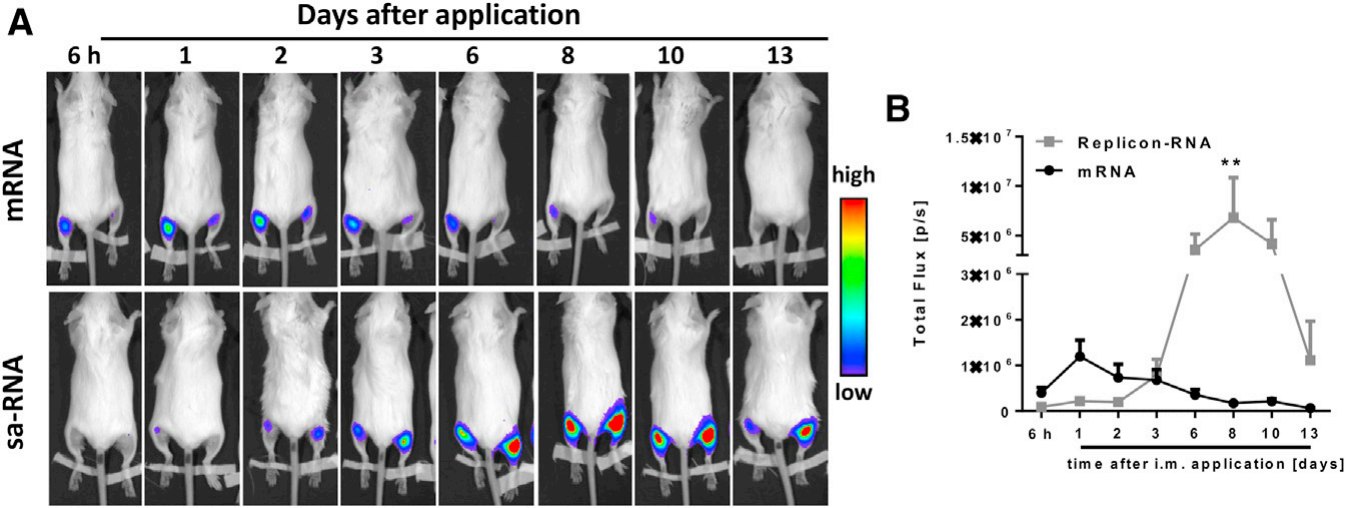
*In Vivo* Imaging of Luciferase Encoded by mRNA and Self-Amplifying RNA BALB/c mice were intramuscularly injected with 4 μg sa-RNA (2 μg per leg) or synthetic mRNA encoding luciferase genes in PBS. At various time points after inoculation, expression was visualized using an IVIS spectrum *in vivo* imaging system after intraperitoneal injection of D-luciferin. One representative image is shown per time point (A). Luciferase levels from n = 6 animals were quantified as relative light units (B). Points represent means ± SEM.

Unlike proteins, nucleic acid vaccines have to be expressed *in situ* prior to inducing an immune response. We investigated whether differences in expression could explain the difference in the dose required for mRNA or sa-RNA immunization. Upon i.m. application of 4 μg mRNA or sa-RNA encoding firefly luciferase, we observed substantial differences between the two RNA vaccine types (Figure 2). Luciferase expression from sa-RNA was delayed, peaking at day 8 after transfer at a 5-fold higher peak level than mRNA and lasting for about 10 days above the peak level of mRNA (Figure 2B).

**Figure 3:**
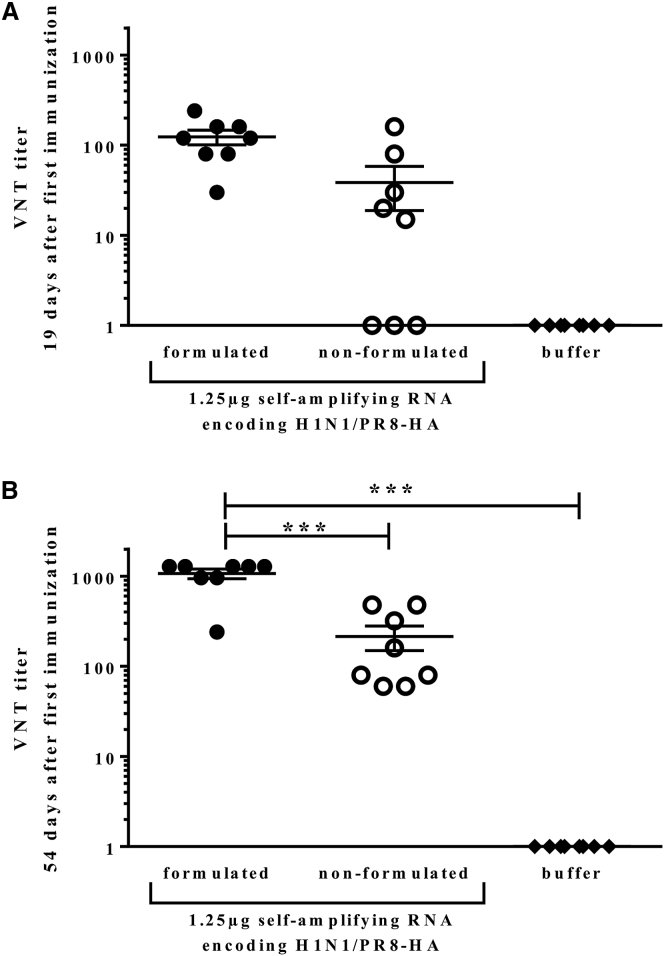
Formulating sa-RNA with PEI Significantly Increases the Antibody Response BALB/c mice were immunized twice, on days 0 and 21, with 1.25 μg PEI-formulated sa-RNA encoding HA or sa-RNA encoding HA alone. Sera was collected at days 19 (A) and 54 (B) and analyzed for influenza virus neutralization. Responses were compared to animals immunized with buffer alone. Points represent individual animals, and lines represent mean of n = 8 animals.

To improve RNA vaccination, different delivery formulation platforms have been described, including polyethylenimine (PEI)-based delivery vehicles.^12^ In our studies, we introduced a medium-length PEI-based formulation suitable for *in vivo* nucleic acid delivery and adapted the formulation to long RNA molecules (data not shown). Next, we analyzed whether this formulation improved sa-RNA vaccination. BALB/c mice were immunized at days 0 and 21 with 1.25 μg sa-RNA encoding the HA of H1N1/PR8 either formulated with PEI or non-formulated (i.e., dissolved in buffer). A third group received only ringer-lactate as a buffer control. All animals immunized with sa-RNA developed an immune response against the HA analyzed by VNT. Both formulated and non-formulated RNA induced VNT response at days 19 (Figure 3A) after the initial vaccination. 54 days after immunization, formulation of sa-RNA encoding the H1N1/PR8-HA resulted in a significantly higher antibody titer compared to using non-formulated sa-RNA (Figure 3B). Taken together, these results demonstrate not only the high potency of sa-RNA-based vaccines but also the potential to improve sa-RNA efficacy by formulation.

### sa-RNA Vaccine Encoding Influenza A Virus HA Protects against Current Seasonal Influenza Strains

**Figure 4:**
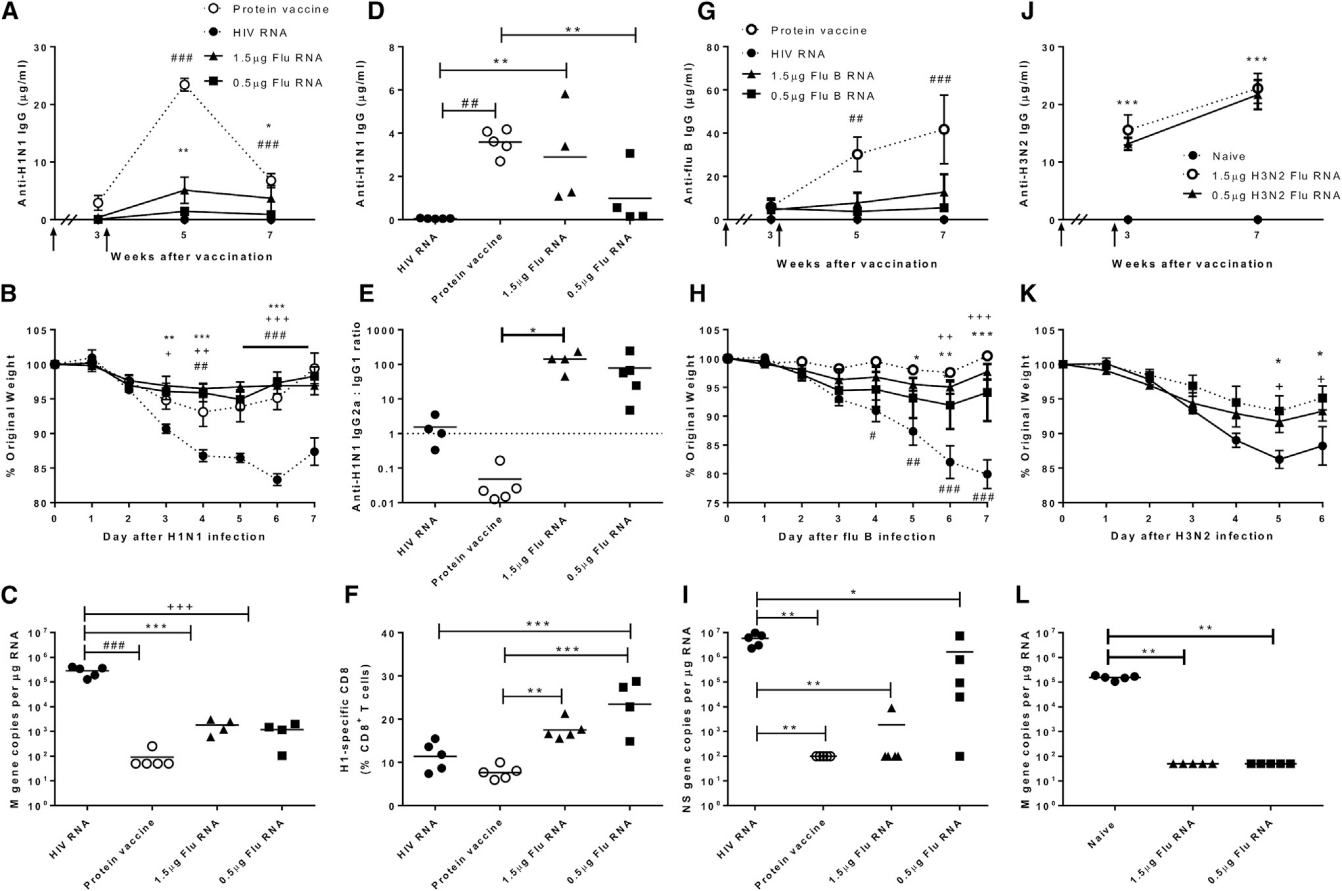
Self-Amplifying RNA Vaccines Are Protective against Seasonal H1N1 and B Influenza Disease and Reduce Viral Load in a Prime-Boost Regime BALB/c mice were i.m. immunized intramuscularly in a prime boost regime with a 3-week interval (indicated by arrows) with 1.5 or 0.5 μg Cal’09 H1N1 HA sa-RNA (A–F), Flu B-Mass (G–I), or X31 H3N2 (J–L). Responses were compared to 1.5 μg HIV gp140 sa-RNA (negative control) or 1.8 μg licensed protein flu vaccine (A–I) or naive animals (J–L). (A) H1N1-specific IgG was measured after vaccination. At 7 weeks, mice were infected intranasally with Cal’09 H1N1 influenza. Weight change was monitored daily (B), and influenza M gene copy number was measured in the lung (C). H1N1-specific total IgG (D) and the ratio of specific IgG2a:IgG1 was measured in serum 4 days after infection (E). (F) H1-specific CD8^+^ T cells were measured in lung tissue on day 7 of infection. (G) For Flu B-Mass-immunized animals, specific IgG was measured by ELISA. (H) At 7 weeks, mice were infected i.n. with B/Florida/06 influenza, and weight change was monitored daily. (I) Influenza B NS gene copy number was measured in the lung. (J) For H3N2-immunized animals, specific IgG was measured by ELISA. (K) At 7 weeks, mice were infected i.n. with X31 H3N2 influenza, and weight change was monitored daily. (L) Influenza A M gene copy number was measured in the lung. Lines and points represent mean of n ≥ 4 mice. *p < 0.05, **p < 0.01, ***p < 0.001 between 1.5 μg flu RNA and negative control; +p < 0.05, ++p < 0.01, +++p < 0.001 between 0.5 μg flu RNA and negative control; and ##p < 0.01, ###p < 0.001 between protein vaccine and negative control.

To test the immunogenicity and efficacy of a sa-RNA vaccine against seasonal influenza virus strains in a mouse model, BALB/c mice received an i.m. prime vaccination followed by a homologous boost 3 weeks later. As a positive control, one group received 1/25^th^ of the human dose of a licensed protein-based seasonal influenza vaccine (Begripal 2014/2015) containing 0.6 μg HA of each A/Califonia/07/2009 (Cal’09 H1N1), A/Texas/50/2012 (Tx50 H3N2), B/Massachusetts/2/2012 (B/Mass). As a negative control another group received 1.5 μg HIV gp140 sa-RNA to exclude unspecific sa-RNA effects leading to an HA-specific immune response. Mice were immunized with either 1.5 or 0.5 μg PEI-formulated sa-RNA encoding Cal’09 H1N1-HA. Serum samples were taken at weeks 3 (immediately prior to boost), 5, and 7 after the priming immunization. Total anti-H1N1 immunoglobulin G (IgG) was determined by ELISA (Figure 4A). In all groups, no IgG could be detected above control levels 3 weeks after the priming dose. After the boost, however, significant levels of anti-H1N1 IgG were measured in mice that received the 1.5 μg HA sa-RNA dose or the protein vaccine (p < 0.01 compared to negative controls). 5 weeks after the prime dose, antibody responses to the licensed protein vaccine were significantly higher than those detected in RNA-immunized mice (p < 0.01), before falling to similar levels by 7 weeks after the prime dose.

To determine whether the sa-RNA vaccine was protective, mice were infected intranasally with 3 × 10^4^ plaque-forming units (PFU) Cal’09 H1N1 4 weeks after the boost. sa-RNA-immunized mice were protected against influenza induced weight loss, with significantly less weight loss from day 3 post-infection compared to the control immunized mice (p < 0.05) (Figure 4B). Influenza M gene RNA was significantly reduced in the lung following both protein and HA sa-RNA vaccination compared with negative control on day 7 after infection (p < 0.01) (Figure 4C), demonstrating reduced viral replication in the animals. H1N1-specific antibody was measured in the serum 4 days after infection, both the protein vaccine and 1.5 μg HA sa-RNA vaccine induced significantly higher total IgG levels than the negative control (p < 0.01) (Figure 4D). Vaccination with protein induced a significantly higher response than low dose sa-RNA (p < 0.01). The ratio of IgG2a:IgG1 H1N1-specific antibodies was significantly different between the protein (mean 0.05) and high dose sa-RNA (mean 141) vaccine (p < 0.05) (Figure 4E), suggesting a Th1-skewed response in the RNA groups. Mice immunized with sa-RNA had a significantly greater proportion of H1N1-specific CD8^+^ T cells in the lungs on d7 after infection than either the protein vaccine or the control group (p < 0.01) (Figure 4F). Taken together, these data demonstrate that H1N1 HA sa-RNA provides effective protection against H1N1 flu challenge, reducing weight loss and viral load as effectively as a protein vaccine despite lower antibody levels, while inducing a higher proportion of specific CD8^+^ T cells.

To check whether the regime described above could be extended to other strains of influenza, the prime-boost study was repeated using a sa-RNA vaccine encoding HA from B/Massachusetts/2/2012 formulated in PEI. The protein vaccine induced significantly higher IgG levels than the HIV RNA control at week 5, furthering increasing by week 7 (Figure 4G). Very little specific IgG was induced in the sa-RNA groups. At week 7, mice were challenged with influenza B/Florida/06 (a Yamagata-like virus antigenically similar to B/Mass), and weight loss was assessed daily. Influenza B sa-RNA provided effective protection against weight loss at both high and low doses from days 5–7 compared with the negative control group (Figure 4H). Influenza-B NS gene RNA was significantly reduced in the lung following both protein and HA sa-RNA vaccination compared with negative control on day 7 after infection (p < 0.05, Figure 4I).

The current seasonal H3N2 is human adapted and only able to bind α2,6-linked sialic acid^13^, ^14^ and therefore not infectious in mice. To test whether an H3N2 antigen was protective we used the HA from X31 (A/Hong Kong/1/68). Immunization with 1.5 or 0.5 μg sa-RNA formulated in PEI induced significantly more X31 hemagglutinin-specific IgG compared with that in control animals (p < 0.001) (Figure 4J). Immunization also significantly reduced weight loss following infection with X31 virus (p < 0.05) (Figure 4K) and reduced viral load at day 7 (p < 0.01) (Figure 4L). Therefore sa-RNA-expressing influenza antigens can protect against matched influenza challenge with 3 different strains of influenza.

### A Trivalent RNA Vaccine Protects against H1N1 Influenza Disease in a Prime-Boost Regime

**Figure 5:**
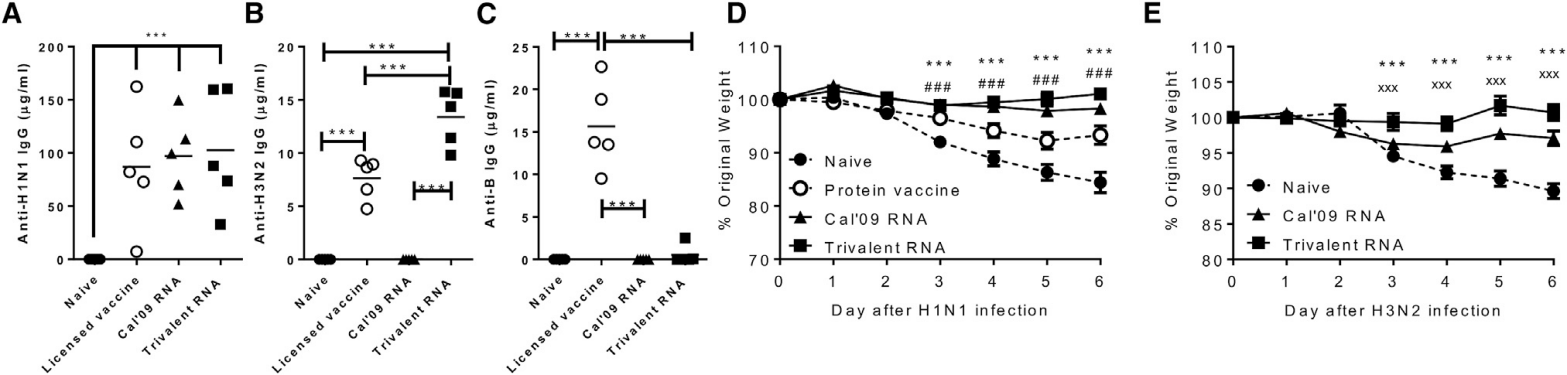
Self-Amplifying RNA Vaccines Are Immunogenic and Protective against H1N1 in Trivalent Combination BALB/c mice were primed i.m. with 1.5 μg each of Cal’09 H1N1, B-Mass, X31 H3N2 HA sa-RNA, 1.5 μg Cal’09 H1N1 sa-RNA alone, or 1.8 μg licensed protein flu vaccine, followed by a homologous boost 3 weeks later. H1N1 (A), H3N2 (B), or Flu B (C) specific antibody was measured by ELISA in sera 7 days after infection. (D) At 7 weeks, mice were infected i.n. with Cal’09 H1N1 influenza, and weight change was monitored daily. (E) 7 days later, the Cal’09 RNA and trivalent RNA groups from the same study were challenged with X31 H3N2 influenza, and responses were compared to new naive controls. (A)–(C) points represent individual animals and lines represent mean. (D) and (E) points represent the mean of n = 5 animals ± SEM. ***p < 0.001 between trivalent sa-RNA and naive; ###p < 0.001 between monovalent sa-RNA and naive; and xxx between monovalent and trivalent RNA (E).

The current influenza vaccine licensed for use—the trivalent protein vaccine used in this study—contains HA from three viral strains. Thus, we wished to test an equivalent trivalent RNA vaccine, to determine whether combining RNA expressed antigens altered immunogenicity. The prime-boost regime used above was adapted such that mice either received 1.5 μg Cal’09 H1N1 HA PEI formulated sa-RNA alone, or a trivalent PEI formulated sa-RNA vaccine containing 1.5 μg each of RNA encoding HA from A/Califonia/07/2009 (H1N1), A/Hong Kong/1/68 (X31, H3N2) and B/Massachusetts/2/2012. Control mice were unvaccinated (naive) or received protein vaccine. Antibody responses against the encoded antigens were measured in sera on day 7 after infection (Figures 5A–5C). Trivalent sa-RNA vaccination induced anti-H1N1 (Figure 5A) and H3N2 (Figure 5B) IgG responses, but only B responses were more inconsistent, with a response in 2 out of 5 animals (Figure 5C), whereas protein vaccination induced antibody responses against all three components. 4 weeks after boost, mice were infected with H1N1 virus, both the single H1 and trivalent RNA vaccines conferred a significant protection from weight loss, from day 4 after infection (Figure 5D). 7 days later, the Cal’09 RNA and trivalent RNA groups from the same study were challenged with X31: new naive controls were used as the initial naive group had not regained weight after the H1N1 challenge and the protein immunisation did not contain an X31 component. The trivalent RNA-immunized animals lost significantly less weight than the Naive or Cal’09-immunized mice (Figure 5E). The Cal’09 group was also partially protected. From this, we can therefore conclude that combining sa-RNA from 3 different HA does not reduce protection against H1N1 or H3N2 challenge compared to immunization with sa-RNA alone.

### A Single Dose of sa-RNA or DNA Vaccine Protects against H1N1 Influenza

**Figure 6:**
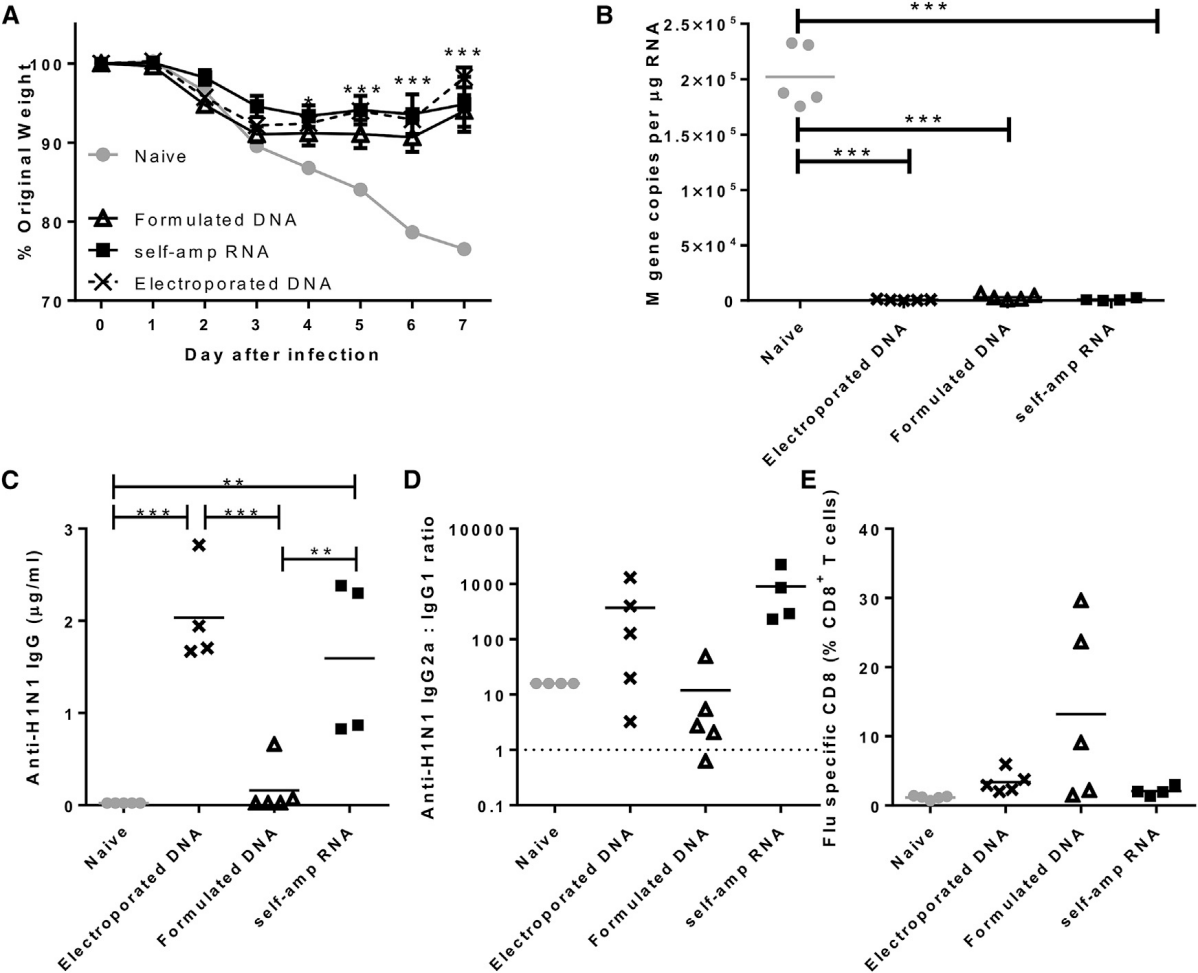
A Single Dose of Self-Amplifying RNA Vaccine Gives Equivalent Protection to Electroporated DNA and Greater Protection than mRNA Encoding the Same Gene (A) BALB/c mice were primed i.m. with 1.5 μg Cal’09 H1N1 as DNA or self-amplifying RNA. RNA was delivered as a formulation; DNA was delivered as a formulation or naked with electroporation. 4 weeks later, mice were infected i.n. with Cal’09 H1N1 influenza, and weight change was monitored daily. (B) M gene copy number was measured in lungs 7 days after infection. 4 days post-infection, H1N1-specific total IgG was measured in serum (C), and the ratio of specific IgG2a:IgG1 determined (D). (E) 7 days post-infection, proportions of flu-specific CD8^+^ T cells were measured in lung tissue by pentamer staining. (A) points represent the mean of n = 5 animals ± SEM. (B)–(E) points represent individual animals, and lines represent mean. *p < 0.05; **p < 0.001, and ***p < 0.001 indicate significance measured by one-way ANOVA.

To determine whether sa-RNA vaccine could elicit “single-shot” immunity, mice received a single shot of Cal’09 H1N1 HA PEI formulated sa-RNA. sa-RNA was compared to other nucleic acid vaccinations: a single 1.5 μg DNA encoding the same gene formulated in the same way as the sa-RNA or 1.5 μg “naked” DNA followed by electroporation as a positive control.^15^ 4 weeks after immunization, mice were infected with H1N1 influenza. Both DNA vaccines and the sa-RNA reduced weight loss after influenza Cal’09 H1N1 infection (p < 0.05) (Figure 6A). Further, when viral load was measured at day 7 post-infection, both DNA vaccines and the sa-RNA significantly reduced viral load, <7,000 copies M gene per 1 μg lung RNA compared with a mean load of 2.02 ± 0.27 × 10^5^ copies/μg in naive mice (p < 0.001) (Figure 6B). Prior to infection, anti-H1N1 IgG levels were measured in the serum (Figure 6C). The sa-RNA and DNA/electro vaccines induced similar amounts of specific IgG (2.03 ± 0.54 and 1.59 ± 0.86 μg/mL, respectively), whereas no other group showed a significant increase above unvaccinated control levels. The sa-RNA vaccine elicited a skew toward IgG2a (mean ratio 904.7) (Figure 6D). Interestingly, the DNA/electroporation-vaccinated group (but not the DNA/PEI group) also showed a skew toward IgG2a, with mean 370.7:1. Influenza-specific CD8 responses were detectable after all vaccinations, but only formulated DNA induced significantly more H1-specific CD8 cells than the naive group (Figure 6E). Thus, a single shot of sa-RNA or DNA encoding HA protects against H1N1 influenza disease, affording protection against weight loss and a significant reduction in viral load.

## Discussion

In this study we have demonstrated that sa-RNA vaccines protect against influenza A or B infection when administered singly or in trivalent combination. Compared to non-amplifying synthetic mRNA vaccines, sa-RNA vaccines induced protection with a 64-fold lower dose, which may result from prolonged and increased transgene expression. Previous studies have demonstrated the efficacy of high-dose mRNA vaccines^4^ and of sa-RNA vaccines against H7N9^16^ and H1N1^11^ influenza. To our knowledge, this is the first study to compare non-amplifying and sa-RNA vaccines expressing the same antigen head to head, it is also the first study to look at a trivalent sa-RNA vaccine for influenza.

We show here that 64-fold less sa-RNA is required to achieve the same level of protection than non-amplifying synthetic -mRNA. On a per-gene basis the dose delivered is even smaller for the sa-RNA group as the sa-RNA construct is larger than the mRNA, 9,300 compared to 2,200 nt. The difference in dose required for protection is important for translation into clinical practice as a significant scale up of the total dose is needed for equivalence in human studies.^2^ Two factors contribute to this increased response per dose, both linked to the replication of the RNA in the host cell: expression and immunogenicity. Based on the luciferase data, expression of antigen from sa-RNA is longer and greater than expression from mRNA (Figure 2). There is also a differential expression profile, with delayed response for the sa-RNA, whether this would change if a larger mRNA dose were used is not clear; other studies have seen longer profiles of luciferase expression after sa-RNA delivery.^17^ The antigen production in sa-RNA-transfected cells is based on the principle of viral replication and therefore results in high antigen expression in transfected cells leading to cellular exhaustion and ultimately cell death of transfected cells. Additionally, due to the prolonged provision of significant amounts of vaccine antigens released from lytic transfected cells provides an ideal constellation for continued B cell stimulation and antibody production. Moreover, sa-RNA as compared to non-amplifying RNA provides additional immune stimuli, e.g., because of generation of double-stranded RNA intermediates and cytopathic effect in transfected cells. *In-vitro*-transcribed RNA is recognized by the host cells by a number of pattern recognition receptors, RNA-dependent protein kinase (PKR),^18^ Toll-like receptors (TLR),^19^ and 2′-5′-oligoadenylate synthetase (OAS),^20^ which will lead to local inflammation. Further studies to separate the relative roles of expression and immunogenicity are of interest in determining the optimum strategy for an RNA vaccine.

One of the aims of the study was to determine whether combining antigens in an RNA vaccine would affect the efficacy of the vaccine. There were two possible causes of interference: inflammation and antigenic sin. It was possible that delivering more RNA would increase inflammation and shut down cellular translation machinery via type I interferon (IFN) and PKR.^21^ However, we did not see any negative effects of increasing dose, either of the sa-RNA or the synthetic mRNA, though it may be that we hadn’t reached the threshold above which the RNA is inhibitory. The other method of interference, antigenic sin, is when expression of structurally similar antigens simultaneously leads to reduced responses to one of them. Sequential DNA vaccination with two different H1 HA antigens has been shown to reduce response to the second antigen,^22^ and was independent of the order in which the mice were exposed to the antigen. Combining two influenza A antigens had no effect on the H1 or H3 response, and trivalent immunized mice were protected against sequentially challenges with H1N1 and H3N2 influenza virus. While the H1N1 response was greater in the trivalent formulation and the H3N2 response was equivalent, the B response was reduced compared to the monovalent immunization. Further work on the dose of the individual antigens for the optimum response in combination is required. The dose of protein vaccine used gave higher levels of antibody response than RNA but it is difficult to compare such different platforms by dose. Ultimately, both types of vaccination were protective, suggesting there is threshold level of antibody and cellular response that can protect against infection and that both protein and RNA vaccination are above this threshold.

The sa-RNA vaccines were also compared to DNA, delivered with electroporation or formulated with PEI. It was of interest because while it induced lower antibody responses, formulated DNA without electroporation was still protective. This protection may have resulted from the H1-specific T cell induction, which was significantly greater after the DNA vaccination; we have shown that DNA vaccine induced CD8 T cells are protective,^23^ and we have previously observed that the route of DNA immunization can change the nature of the immune response while not changing the level of protection.^15^ DNA vaccines, have been highly effective in small animal models, but this pre-clinical success has not translated into clinical success.^2^ One approach that has improved DNA vaccination efficacy in clinical settings is the use of electroporation^24^ and electroporation can also significantly enhance the antibody response to sa-RNA-expressing influenza hemagglutinin.^25^ While it has been shown to be tolerable in clinical trial settings, immunization with electroporation is more painful than immunization without it.^24^, ^26^ It also requires trained operatives to deliver, a power source and specialist equipment to deliver, which means that while of value as an investigative tool, it is unlikely to be translated into broader clinical practice. In contrast, mRNA vaccines have been already shown to be successfully applied to larger animals and entered clinical trials without requiring electroporation for delivery.^10^, ^27^ The sa-RNA combines ideal immunological and biopharmaceutical properties and are therefore an attractive alternative. The data presented here supports the further development of sa-RNA for preventative vaccine usage.

## Materials and Methods

### RNA Synthesis by *In Vitro* Transcription

T7 *in vitro* transcription is based on protocols provided by the MEGAscript T7 Transcription Kit (Thermo Fisher, formerly Ambion). The general procedure starting with linear DNA template containing the T7 promoter, and particularly with respect to co-transcriptional capping with the synthetic cap analog beta-S-ARCA(D1) (used in 4:1 ratio regarding guanosine triphosphate [GTP]), is carried out similarly to as described before.^5^ Based on previous work, e.g., Pokrovskaya and Gurevich,^28^ high-yielding processes qualified for our particular systems were developed; here, protocols have been modified and optimized with respect to long sa-RNA with up to 10,000 nt.

#### Intramuscular Injections and *In Vivo* Bioluminescence Imaging

Mice were anesthetized by inhalation anesthesia (isoflurane 2.5%) (Abbott, Ludwigshafen, Germany). Subsequently, 20 μL of pre-mixed RNAs in RNase-free PBS (Life Technologies, Darmstadt, Germany) was injected i.m. to the *tibialis posterior*. Following intraperitoneal (i.p.) injection of 100 mg/kg body weight D-luciferin (PerkinElmer, Rodgau, Germany), inhalation anesthesia (isoflurane 2.5%) was introduced, and, during maintenance, serial images of the animals were taken at the indicated time points using an IVI Spectrum imaging system (PerkinElmer). Photons emitted were collected for 1 min. Bioluminescence intensity from the muscle region of interest was quantified using Living Image software (PerkinElmer).

### VNT

To determine the level of neutralizing antibodies against HA in the serum of animals, VNTs were performed in accordance with the Manual for the Laboratory Diagnosis and Virological Surveillance of Influenza (WHO Global influenza Surveillance Network). A serial dilution of serum samples starting with 1:10 was incubated for 2 hr with 100 TCID_50_ of infectious influenza virus. The final serum dilution of this assay was 1:1,280 and thereby also the upper detection limit. The serum-virus mix was then applied to confluent Madin-Darby canine kidney (MDCK) monolayer in 96-well plates and incubated for another 3 days. 50 μL of supernatant was thereafter incubated with 50 μL of 0.5% chicken red blood cells (Lohmann Tierzucht, Cuxhaven, Germany), and red blood cell agglutination was evaluated. The VNT titer was recorded as the inverse of the lowest dilution that inhibited agglutination (VNT/50 μL).

### HAI Assay

To determine the serum level of anti-HA antibodies that inhibit hemagglutination in mice, sera were collected and HAI assay was performed following the Manual for the laboratory diagnosis and virological surveillance of influenza (WHO Global influenza Surveillance Network). Briefly, serum samples were treated overnight with receptor destroying enzyme II “Seiken” in a 1:5 ratio (RDE [II], Denka Seiken, Japan) followed by heat inactivation for 30 min at 56°C. Sera were used in duplicates and serial dilutions (1:2) were performed before adding 4 hemagglutinating units (HAUs) of H1N1/PR8 virus. After 60 min incubation at room temperature, 50 μL of 0.5% red blood cells (Lohmann Tierzucht) were added and the mixture incubated for 30 min at room temperature before evaluation of agglutination. The HAI titer was recorded as the inverse of the lowest dilution that inhibited agglutination (HAI/50 μL).

### Mouse Immunization and Infection

6- to 8-week-old female BALB/c mice were obtained from Harlan UK (Exelby, UK) or Janvier (Genest Saint Isle, France) and kept in specific pathogen-free (SPF) conditions in accordance with the German animal welfare law and United Kingdom’s Home Office guidelines. All work was approved by the Animal Welfare and Ethical Review Board (AWERB) at Imperial College London or by the local animal welfare committee of Rhineland-Palatinate (reference number G-13-8-063). For the inactivated virus control group, mice were immunized with 10 μg per 20 μL H1N1/PR8 virus (Charles River, Wilmington, MA, USA), and for licensed protein vaccine groups, mice were immunized i.m. via the *anterior tibialis* with 20 μL Begripal 2014/15 (Novartis Vaccines) containing 0.6 μg HA from each of B/Massachusetts/2/2012, A/Texas/50/2012 (H3N2)-like, and A/California/7/2009 (H1N1) pdm09-like viruses. For synthetic RNA vaccination, mice were injected i.m. with 20 μL non-formulated mRNA, non-formulated, or formulated sa-RNA. For DNA vaccination, mice received 1.5 μg formulated DNA or naked DNA with electroporation i.m. Where used, two lots of 5 pulses of 150 V with switched polarity between pulses were delivered using a CUY21 EDIT system (BEX, Japan). For infections, mice were either anesthetized with ketamin-rompun before infected intranasally (i.n.) with 30 μL containing 2.4 × 10^4^ PFU (10x MLD_50_) of H1N1/PR8 or anesthetized using isoflurane followed by i.n. application with 3 × 10^4^ PFU A/California/7/2009 (H1N1) influenza.

### Influenza

H1N1 influenza virus A/Puerto Rico/9/1934 was a kind gift from Veronika von Messling (Paul-Ehrlich Institute, Langen, Germany) and thereafter grown in-house on MDCK cells in fetal calf serum (FCS)-free minimum essential media (MEM) (ThermoFisher Scientific Life Technologies, Darmstadt, Germany). H1N1 influenza (strain A/England/195/2009), isolated by Public Health England in the United Kingdom, April 2009,^29^ and influenza (strain B/Florida/4/06) isolated in the United States in 2006, were grown in MDCK cells, in serum-free DMEM supplemented with 1 μg/mL trypsin. The virus was harvested 3 days after inoculation and stored at −80°C. Viral titer was determined by plaque assay as previously described.^30^

### Semiquantitative Antigen-Specific ELISA

Antibodies specific to influenza H1N1 were measured in sera using a standardized ELISA. MaxiSorp 96-well plates (Nunc) were coated with 1 μg/mL H1N1 surface protein or a combination of anti-murine lambda and kappa-light-chain-specific antibodies (AbDSerotec, Oxford, UK) and incubated overnight at 4°C. Plates were blocked with 1% BSA in PBS. Bound IgG was detected using horseradish peroxidase (HRP)-conjugated goat anti-mouse IgG (AbD Serotec). Alternatively, IgG1 or IgG2a were detected using subtype-specific secondary antibodies. A dilution series of recombinant murine immunoglobulin was used as a standard to quantify specific antibodies. 3,3',5,5'-Tetramethylbenzidine (TMB) with H_2_SO_4_ as stop solution was used to detect the response, and optical densities were read at 450 nm.

### Tissue and Cell Recovery and Isolation

At specified time points post-immunization, blood samples were taken by tail-vein bleed, and sera were isolated after clotting by centrifugation. Mice were culled using 100 μL intraperitoneal pentobarbitone (20-mg dose; Pentoject, Animalcare, UK), and tissues were collected as previously described.^31^ Blood was collected from carotid vessels, and sera were isolated after clotting by centrifugation. Lungs were removed and homogenized by passage through 100-μm cell strainers and then centrifuged at 200 × *g* for 5 min. Supernatants were removed, and the cell pellet treated with red blood cell lysis buffer (ACK; 0.15 M ammonium chloride, 1 M potassium hydrogen carbonate, and 0.01 mM EDTA [pH 7.2]) before centrifugation at 200 × *g* for 5 min. The remaining cells were resuspended in RPMI 1640 medium with 10% fetal calf serum, and viable cell numbers were determined by trypan blue exclusion.

### Influenza Viral Load

Viral load *in vivo* was assessed by Trizol extraction of RNA from frozen lung tissue disrupted in a TissueLyzer (QIAGEN, Manchester, UK). RNA was converted into cDNA, and qRT-PCR was carried out using bulk viral RNA for the influenza M gene and mRNA using a 0.1-μM forward primer (5′-AAGACAAGACCAATYCTGTCACCTCT-3′), a 0.1-μM reverse primer (5′-TCTACGYTGCAGTCCYCGCT-3′), and a 0.2-μM probe (5′-FAM-TYACGCTCACCGTGCCCAGTG-TAMRA-3′) on a Stratagene Mx3005p (Agilent Technologies, Santa Clara, CA, USA). M-specific RNA copy number was determined using an influenza M gene standard plasmid.

### Flow Cytometry

Cells were stained with Fixable Violet Dead Cell Stain (Life Technologies, UK), washed, suspended in Fc block (Anti-CD16/32, BD) in PBS-1% BSA, and then stained with the following surface antibodies: influenza A H1 HA_533-541_ IYSTVASSL Pentamer R-PE (Proimmune, Oxford, UK), CD3-FITC (BD, Oxford UK), CD4- PE/Cy7 (BioLegend, CA, USA), and CD8-APC-H7 (BD). Analysis was performed on an LSRFortessa Flow Cytometer (BD). Fluorescence minus one (FMO) controls were used for surface stains.

#### Statistical Analysis

Calculations described in the figure legends were performed using Prism (v.6) (GraphPad Software, La Jolla, CA, USA).

## Author Contributions

L.L., E.K., A.B.V., S.E., D.B., and K.C.R. performed the studies; T.B., M.P., H.H., and L.W. developed and provided the RNA vaccine constructs; A.B.V., U.S., and J.S.T. wrote the paper; and J.S.T., S.T.R., and A.B.V. designed the studies.

## Conflicts of Interest

A.B.V., S.E., H.H., K.C.R., L.W., M.P., T.B., S.T.R., and U.S. are or were employees of Biontech or related companies. All other authors declare no competing financial interests.
